# Isolated Intraocular Relapse of Pediatric B-cell Precursor Acute Lymphoblastic Leukaemia Following Chimeric Antigen Receptor T-lymphocyte Therapy

**DOI:** 10.7759/cureus.10937

**Published:** 2020-10-14

**Authors:** Delphine Veys, Alice Norton, John R Ainsworth, Persis Amrolia, Giovanna Lucchini

**Affiliations:** 1 Hematology, Addenbrookes Hospital, Cambridge University Hospitals Foundation Trust, Cambridge, GBR; 2 Hematology, Birmingham Women’s and Children’s National Health Service Foundation Trust, Birmingham, GBR; 3 Ophthalmology, Birmingham Children’s Hospital, Birmingham, GBR; 4 Pediatrics, Great Ormond Street Hospital for Children National Health Service Foundation Trust, London, GBR

**Keywords:** car-t, b-all, intraocular relapse, extramedullary disease

## Abstract

Chimeric antigen receptor T-lymphocytes (CAR T) targeting the CD19 surface antigen have achieved a breakthrough in the treatment of multiply relapsed and refractory bone marrow (BM) disease in childhood B-cell precursor acute lymphoblastic leukaemia (B-ALL). The ability of CAR T therapy to treat extramedullary (EM) disease is less proven. However, early reports suggest trafficking of CART-cells to the central nervous system (CNS) as well as other EM sites. We describe a case of isolated intraocular relapse of pediatric B-ALL following CAR T-cell therapy, which had successfully controlled multiply relapsed BM and CNS disease. CAR T-cells may not be able to traffic into the eye, making it a “sanctuary” site during therapy.

## Introduction

Chimeric antigen receptor T-lymphocytes (CAR T) targeting the CD19 surface antigen have achieved a breakthrough in the treatment of multiply relapsed and refractory childhood B cell precursor acute lymphoblastic leukaemia (B-ALL). Complete response rates between 60% and 90%, with a one-year event-free survival (EFS) up to 50% have been reported [1-3]. Data are largely derived from patients with bone marrow (BM) disease, and experience using CAR T therapy in pediatric patients with extramedullary (EM) disease is limited. Patients with EM disease have largely been excluded from clinical trials due to concern over increased neurotoxicity risk, especially in CNS disease, the most common EM site. Nevertheless, small studies have shown that CAR T- cells can reach and treat CNS [4] and non-CNS sites such as cervix, breast, bone, and testes [5,6]. However, not all EM disease may be accessible to CAR T-cells. We report a case of intraocular EM relapse despite successful eradication of BM and CNS disease following CAR T therapy.

## Case presentation

A nine-year-old boy presented in July 2014 with lethargy, intermittent fever and leg pain such that he could no longer walk. He was noted to be pancytopenia (haemoglobin 87g/L, white blood count (WBC) 5.5 x109/L, neutrophils 0.5x109/L, platelets 73x109/L) with blasts circulating in peripheral blood. A BM was hypercellular with 94% blasts, phenotypically marked as CD19, 22, 34, 79a, Human Leukocyte Antigen - DR isotype (HLA-DR) and Terminal deoxynucleotidyl transferase (TdT) positive. Fluorescence in situ hybridisation (FISH) showed a t(12;21) ETV6 RUNX1 translocation in 90% of interphases. The cerebrospinal fluid (CSF) was clear. He was diagnosed as having B lineage acute lymphoblastic leukaemia (ALL) and entered the UK Acute Lymphoblastic Leukaemia trial 2011 (UKALL 2011). He received regimen A induction and switched to post-induction regimen C due to risk of minimal residual disease (MRD) by Immunoglobulin (Ig) and T-cell receptor (TCR) (Ig/TCR) at the end of induction. Consolidation was complicated by pancreatitis presumably secondary to asparaginase.

In January 2017, two and a half years after diagnosis, he developed right arm bone pain. A BM aspirate was effaced with 100% blasts with the same phenotype as at presentation. An early BM relapse (CSF clear) was diagnosed, and he received Clofarabine/Cyclophosphamide/Etoposide. He next received a matched sibling donor combined BM and cord blood transplant, conditioned with total body irradiation (TBI) 1440 cGy + Etoposide 60 mg/kg in April 2017. He engrafted with 100% donor chimerism. However, his post-transplant course was complicated with a lower respiratory tract infection which eventually improved with empiric therapy. However, he was left with an oxygen requirement. He developed grade I acute graft-versus-host disease (GVHD) of the skin and an episode of adenoviraemia which settled with cidofovir therapy. His BM was MRD negative in July 2017, and he stopped all immunosuppression by August 2017.

In April 2018 he developed hyperphagia, weight gain, somnolence, lethargy, shortness of breath on exertion, and myalgia together with depressed mood. Thyroxine was commenced for hypothyroidism. However, his symptoms persisted, and he developed headaches with blurred vision but no vomiting. A BM in May 2018 was hypocellular and in morphological CR but with a borderline molecular MRD 1X10-5. The CSF had a WBC of 3910 cells/mm3 with blasts on the cytospin, and immunophenotype on these showed 86% of the cells to be CD19 positive precursor B-cells, confirming an isolated CNS relapse. He was treated with a twice-weekly triple (methotrexate, cytosine arabinoside, steroids) intrathecal (IT) with, with a clearance of CSF by June 2018. Systemically, he received four doses of vincristine without dexamethasone and subsequent maintenance therapy of 6-mercaptopurine and methotrexate from August 2018. 

At this point, following multiple disease relapses, he was eligible for treatment with chimeric antigen receptor (CAR) T-cells, and he was enrolled on a CAR T-cell study: ‘Immunotherapy with CD19 CAR redirected T-cells for high risk/relapsed paediatric CD19+ acute lymphoblastic leukaemia and other haematological malignancies’ (CARPALL ClinicalTrials.gov number NCT03131934). He underwent leukapheresis in December 2018, and autologous CD19 CAR T-cells were generated. He was initially admitted for CAR-T therapy at the beginning of January 2019 but was symptomatic with a metapneumovirus on nasopharyngeal aspirate (NPA), and treatment was delayed. Unfortunately mid-January he suffered asymptomatic CNS relapse and was treated with further ITs (receiving in total 23 ITs since relapse post-transplant). By the end of February, the CSF showed WBC <1 cells/mm3 but with possible blasts on the cytospin (CNS2), and the BM was flow and molecular MRD negative. He was readmitted in March 2019 but spiked a fever on admission and had a cough. Extended panel NPA was negative, and computerised tomography (CT) chest scan did not show infiltrates but the progression of known chronic chest lesions. He underwent bronchoalveolar lavage (BAL) which detected H1N1 influenza and was treated with oseltamivir and then switched to zanamivir. Cranial Magnetic Resonance Imaging (MRI) performed as baseline screening showed perioptic thickening. The patient reported right eye discomfort. He was reviewed by the ophthalmology team and found to have the right eye decreased visual acuity and colour vision associated with chorioretinal atrophy, peripheral pigmentary change, but no optic disc oedema or inflammation. The opening pressure on lumbar puncture was slightly raised at 35 cms H2O suggestive of intracranial hypertension but without clinical symptoms. Findings were considered to reflect chronic changes from previous treatment and/or infection and not ocular relapse. 

By mid-March he was able to proceed with immunotherapy, receiving a reduced dose of fludarabine 120mg/m2 in view of renal impairment, and cyclophosphamide 1.5g/m2 as lymphodepletion followed by 1 x 106 autologous CD19 CAR+ T-cells/kg. Following his cell infusion, he developed cytokine release syndrome grade 1, which settled spontaneously. He did not show neurotoxicity. A BM performed one month post CAR T-cell therapy was hypocellular, but the flow and molecular MRD were negative. CSF was clear. As described, he had persistent neutropenia requiring GCSF up to 4 months post CAR T-cell administration.

**Figure 1:**
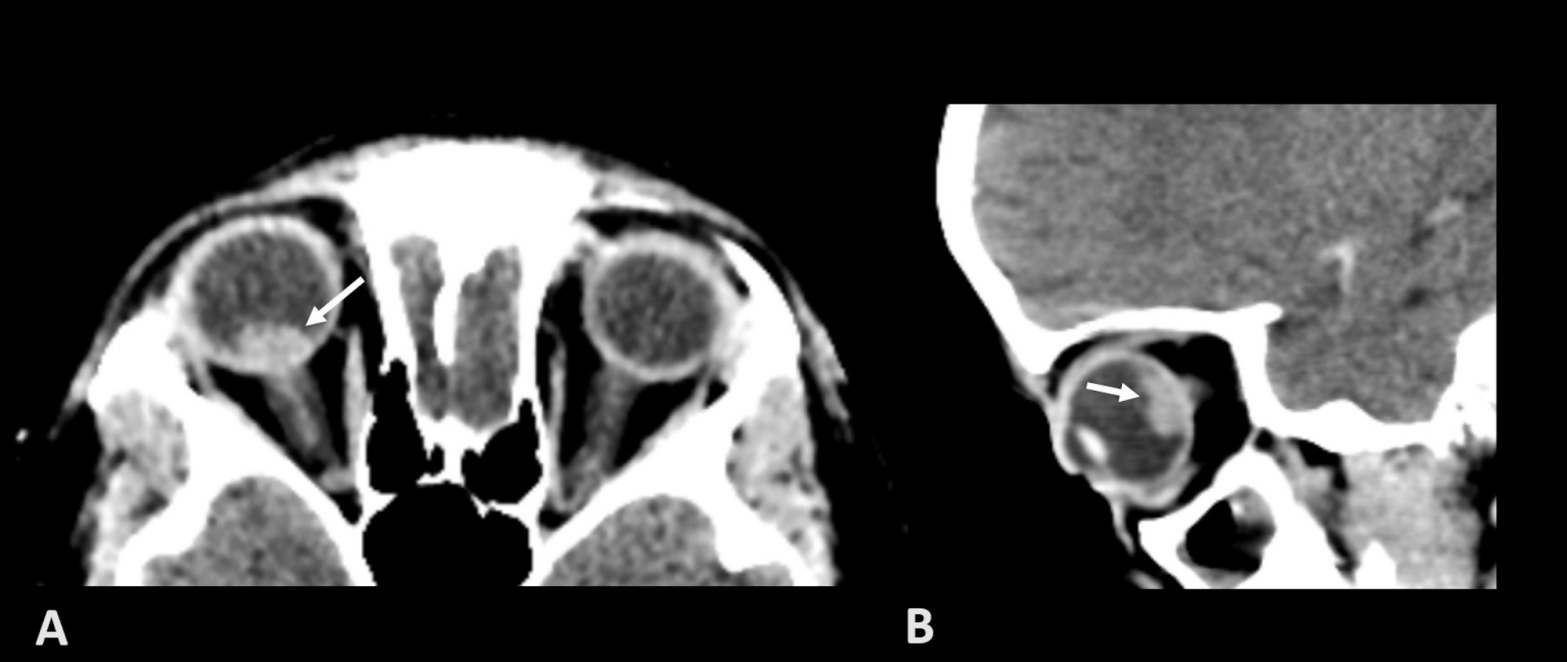
Axial (A) and Sagittal (B) post-contrast CT of the orbits shows a spontaneously hyperdense mass in the posterior aspect of the right globe (white arrows).  The left eye is unremarkable.

Between five-six months post CAR T-cell therapy, he experienced multiple chest infections and neutropenic fevers. He was treated with broad-spectrum antibiotics and started on immunoglobulin replacement therapy, with clinical improvement. A chest CT performed in August showed left lower lobe nodular lesions accompanied by a left pleural effusion, which was tapped and cultures grew Aureobasidium (a fungal mould). He responded well to the combination of antifungal treatment with ambisome and voriconazole and was discharged home in September 2019 with no chest sequelae. However, during this admission, he experienced a period of agitation which prompted neurological investigations including a cranial CT scan, which documented a 7 mm round, enhancing lesion in the right eye (figure 1). Right eye visual acuity was reduced, unnoticed by the patient. MRI head confirmed the findings and absence of intracranial lesions. A vitrectomy with tumour and vitreous biopsy documented relapse of CD19 positive leukaemia. Disease restaging showed clear CSF; BM was in morphological and MRD remission. Importantly, CAR T-cells were still present in the peripheral blood and BM, and he had persistent B cell aplasia. Hence this was an isolated intraocular EM relapse. Whilst targeted radiotherapy was considered, after multidisciplinary discussion with patient and family, uncomplicated surgical enucleation with 22mm medpor primary reconstruction and peroperative cosmetic shell was undertaken. The primary indication was to maximise the chance of re-establishing full disease remission. Histology revealed 10mm optic nerve invasion including pia mater, demonstrating either intraocular relapse had invaded the optic nerve extensively, or growth from optic nerve into the eye. Fortunately, a snare was utilised at surgery, so achieving a 4mm tumour-free margin.

Given the persistence of systemic CAR T-cells and CNS negative status, systemic chemotherapy was not given, and disease surveillance was maintained systemically and in CSF by persisting CAR T-cells. At latest follow-up, 18 months post CAR T-cell therapy and 13 months since surgery, he remains well, with normal blood counts, good T-cell reconstitution, and free of disease in the CNS, BM, orbit and fellow eye. He remains B cell aplastic due to CAR T-cell persistence and continues on subcutaneous immunoglobulin replacement.

## Discussion

Relapse of childhood ALL in the eye is rare, accounting for around 2% of relapses [7]. Intraocular ALL relapse may occur either alone or in combination with another site [8,9]. Curative approaches generally employ full systemic chemotherapy relapse protocols together with targeted therapy (e.g. local radiotherapy) to the affected eye, as further systemic or local relapse is likely. There is no published data on the trafficking of CAR T-cells into the eye. Anti-CD22 CAR T-cells are trafficking to the site of orbital relapse in one case [10]. In our case report, CD19 positive intraocular relapse occurred in the absence of disease elsewhere and in the presence of circulating CAR T-cells suggesting anti-CD19 CAR T-cells could not traffic into the eye sufficiently.

Further data will be required to see if this is specific to this anti-CD19 CAR construct or other CAR constructs alike. The patient responded well to targeted therapy alone, in this case, enucleation, remaining in complete remission for over one year. This suggests that the eye is a “sanctuary” site from CAR T-cells which have nevertheless maintained disease control in the BM and CNS. Physicians need to be aware that intraocular relapse of B-ALL may occur despite successful CAR T-cell responses in the bone marrow and CNS, and additional local therapy may be required to the eye. The pre-existent eye changes, in this case, may represent a previously unrecognised site of relapse that had achieved macroscopic regression during treatments before CAR T-cell therapy. It is more likely that ocular relapse occurred from residual intraocular leukaemic cells undetectable by current techniques, rather than leukemic cells entering the eye whilst the patient was in full remission following CAR T-cell therapy. If so, then it may be possible to recognise patients at risk by eye examination before CAR T-cell therapy. Also, intraocular relapse may be predicted to occur early, and a limited period of targeted screening following successful CAR T-cell therapy may be sufficient. 

## Conclusions

CAR T-cell therapy appears to be curative with multiply relapsed or refractory B-ALL in the BM, possibly treating relapse of B-ALL in the CNS and certain other EM sites. However, at least with this CAR construct the eye may be a “sanctuary” site from CAR T-cell trafficking, and additional targeted treatment may be required in the presence of intraocular or optic nerve disease. Prolonged remission may be re-established with prompt definitive local treatment.
